# The impact of perceived gingival recession on oral health-related quality of life: a cross-sectional study of Saudi adults

**DOI:** 10.3389/froh.2026.1878291

**Published:** 2026-07-15

**Authors:** Marwa Madi, Sarah Fita, Hoda Albaqawi, Mohammed Albander, Muntathir Alahmed, Gillan I. El-Kimary, Ahmed Elakel, Eman Aljoghaiman

**Affiliations:** 1 Department of Preventive Dental Sciences, College of Dentistry, Imam Abdulrahman Bin Faisal University, Dammam, Saudi Arabia; 2 College of Dentistry, Imam Abdulrahman Bin Faisal University, Dammam, Saudi Arabia; 3 Department of Preventive Dental Sciences, College of Dentistry, King Faisal University, Al-Ahsa, Saudi Arabia

**Keywords:** dentine hypersensitivity, gingival recession, OHIP-14, oral health-related quality of life, Saudi Arabia

## Abstract

**Background/objectives:**

Gingival recession (GR) is a common periodontal condition that can lead to both aesthetic and functional concerns. There is limited data on their impact on patient-reported outcomes in Saudi Arabia. This study aimed to evaluate the impact of self-reported gingival recession on oral health-related quality of life (OHRQoL) among adults in Saudi Arabia.

**Materials and methods:**

A cross-sectional study was conducted among 619 adults in Saudi Arabia. Data was collected using a validated, self-administered questionnaire covering demographic characteristics, oral hygiene practices, and the Oral Health Impact Profile-14 (OHIP-14) to assess seven dimensions of OHRQoL. Statistical analysis included Chi-square tests and logistic regression models adjusted for age, gender, and socioeconomic status.

**Results:**

The prevalence of perceived GR was significantly higher in males com-pared to females (30.4% vs. 22.7%; *p* = 0.0295) and increased progressively with age (*p* = 0.0001). GR was significantly associated with all 14 items and seven domains of the OHIP-14. The most frequent complaints included painful aching (84.5%) and difficulty eating (76.4%). The strongest associations were found in psychological domains, with high levels of self-consciousness (59.9%) and embarrassment (61.4%) reported by those with recession. Participants with GR were significantly more likely to have a “poor” overall OHIP score (≥35) compared to those without recession (14.6% vs. 2.9%; *p* < 0.0001). Adjusted logistic regression confirmed that GR was a persistent independent predictor of poorer quality of life across all functional, physical, and psychosocial domains (*p* < 0.05).

**Conclusions:**

Self-reported gingival recession exerts a considerable negative impact on the quality of life of adults in Saudi Arabia, particularly regarding physical pain and psychosocial well-being. These findings emphasize the importance of improving awareness of gingival recession and promoting early clinical intervention to reduce its functional and psychosocial burden. Recession was self-reported and not clinically verified, so the reported associations should be interpreted with this limitation in mind.

## 1 Introduction

Gingival recession is defined as the apical shift of the gingival margin beyond the cementoenamel junction (CEJ) (1). This multifactorial condition is highly prevalent (2, 3). A global systematic review reported a pooled prevalence of 75.42% for buccal recession (2). In a systematic review of cross-sectional prevalence studies, Marschner et al. reported prevalence rates of 81.1%, 48.4%, and 16.2% for gingival recession depths of ≥1 mm, ≥3 mm, and ≥5 mm, respectively (3). These figures are derived from direct clinical periodontal assessments. In contrast, studies relying on self-reported perception of gingival recession typically report lower prevalence estimates.

This mucogingival deformity is multifactorial (4, 5). According to the narrative review by Cortellini and Bissada (5) in the AAP 2017 World Workshop classification, predisposing factors to gingival recession include gingival biotype and attached gingiva, improper tooth brushing, cervical restorative margins, orthodontic movement, and persistent gingival inflammation despite treatment due to factors hindering access for oral hygiene, such as shallow vestibule and high frenal attachment (5).

In addition to the high prevalence of gingival recession, a systematic review by Chambrone and Tatakis (6) found that, if left untreated, gingival recession in subjects with good oral hygiene tends to progress over time. While some individuals may remain asymptomatic, others might experience discomfort, tooth sensitivity, concerns about tooth loss, or aesthetic issues (1). In addition, exposed root surfaces can lead to plaque accumulation and gingival bleeding (1).

Previous research has demonstrated associations between oral diseases and quality of life (7). In this regard, some observational studies have found associations between periodontal disease parameters and oral health-related quality of life OHIP-14 in different settings (8, 9), indicating that this disease is associated with a negative impact on quality of life. Using a random sample of Australian individuals, Brennan et al. (9) observed that GR of 6 mm was associated with worst general quality of life.

Studies on gingival recession and its effect on oral health-related quality of life in Saudi Arabia are limited. Given the high prevalence of gingival recession worldwide and its potential esthetic and functional consequences, it is important to better understand the impact of this condition on patients' quality of life. Despite the limited evidence available from Saudi Arabia, few studies have investigated the relationship between gingival recession (GR) and oral health-related quality of life (OHRQoL) within this population. Although symptoms such as dentine hypersensitivity may accompany gingival recession, the effect of gingival recession on oral health-related quality of life has not been adequately explored in the Saudi context. Therefore, the aim of this study was to evaluate the impact of gingival recession on oral health-related quality of life among adults in Saudi Arabia.

## 2 Materials and methods

This study employed a cross-sectional design using a self-reported questionnaire to assess the consequences and impact of gingival recession (GR) and dentine hyper-sensitivity on the quality of life among adults in Saudi Arabia. The study was approved by the Institutional Review Board (IRB) of Imam Abdulrahman Bin Faisal University (2022-02-372). Participants were informed about the study's purpose, and a written consent was obtained before participation. Data was anonymized to ensure confidentiality. The study was conducted from September 2023 to December 2024. Based on a 95% confidence level and a 5% margin of error, the minimum required sample size was 384 participants. The final sample comprised 619 participants, including 165 individuals with perceived gingival recession, exceeding recommended event-per-variable requirements for the adjusted regression analyses and supporting adequate statistical power to detect the observed associations.

### 2.1 Study participants

The study included participants from all genders from diverse demographic backgrounds in Saudi Arabia. A convenience sample was recruited through dental clinics, community centers, and online platforms across multiple regions of Saudi Arabia, and the questionnaire was self-administered online through a single distributed electronic form. All 619 analyzed responses were collected through this online form, and the response rate was approximately 90% of the invitation links sent. Because data collection was conducted entirely online, no comparison between online and in-person responses was applicable. Incomplete questionnaires were excluded prior to analysis. Inclusion criteria required participants to be aged 18 years or older and able to complete the questionnaire in Arabic or English. Exclusion criteria included individuals with cognitive impairments or those unwilling to provide informed consent.

### 2.2 Study questionnaire

The first questionnaire was designed to collect data on demographic characteristics, oral hygiene practices, GR and Dentine hypersensitivity related factors (10). Dentine hypersensitivity items were collected as part of this broader protocol and were analyzed and reported in our previous publication (10); they were not a separate outcome of the present analysis, which focuses on gingival recession and oral health–related quality of life. Dentine hypersensitivity is referred to as a clinically associated condition that may contribute to the pain-related impacts captured by the OHIP-14. The impact of periodontal diseases on quality of life (QoL) was assessed using the previously validated English and Arabic versions of the Oral Health Impact Profile-14 (OHIP-14) (11, 12); the Arabic instrument was the culturally adapted and validated version reported by Al Habashneh et al. (12), and no new translation was undertaken for the present study. Prior to commencing the questionnaire, participants were provided with a brief explanation of gingival recession in plain language, accompanied by a standardized clinical photograph showing a clearly recognizable recession, to support self-identification of the condition. Recession status was therefore determined entirely by participant self-report; no intra-oral examination or periodontal charting was carried out, and no subsample was clinically validated against examiner findings. The OHIP-14 is a self-administered questionnaire consisting of 14 items designed to evaluate QoL across seven dimensions: functional limitation, physical pain, psychological discomfort, physical disability, psychological disability, social disability, and handicap (Supplementary File S1). Each dimension is assessed using two items. The instrument retains its original, validated seven-domain structure (13). For descriptive purposes in the present study, and to reflect the everyday burden of gingival recession and its commonly associated conditions, the 14 items were additionally summarized under three broader, clinically oriented categories:

(a) The effect of any oral disease including gingival recession and/ or dentine hypersensitivity on the daily routine quality of life; this domain included 8 questions about their effect on taste perception, dietary habits and pronunciation of words.
(b) The presence or absence of pain or any unrelaxing or irritable condition that the patient would be subjected to daily and could affect his functional ability; this domain included 4 questions
(c) The effect of any oral disease, gingival recession, dentine hypersensitivity and their consequences on the general psychological status of the patient; this domain included 2 questions. This three-category grouping was used only as a descriptive framework to aid interpretation; all statistical analyses were conducted at the level of the 14 individual OHIP-14 items and the validated total score, not on these summary categories.

Participants were asked to indicate how often they experienced these negative impacts over the previous 12 months.

Responses were recorded on a five-point Likert scale ranging from 0 (never) to 4 (very often). Item scores were summed up to generate an overall OHIP-14 score, with higher scores indicating poorer quality of life. The maximum possible total OHIP-14 score was 56 (14 × 4). For descriptive and analytical purposes, overall OHIP was further categorized as good when the total score was <60% of the maximum possible score (<35) and poor when the score was ≥60% (≥35). This dichotomy is a pragmatic cut-point rather than a universally validated threshold; the same classification (good <35, poor ≥35) has been applied previously in a Saudi adult population by Thirunavukkarasu et al. (14). The categorization was used only as a summary measure, while the primary analyses relied on the individual items and the continuous total score.

The questionnaire was validated for content and clarity by a panel of three dental experts and piloted on a small sample (*n* = 30) to assess content validity within the target population. Participants were asked to identify any difficulties in understanding the questionnaire items to evaluate comprehensiveness. Necessary modifications were made during this pilot phase to enhance clarity and intelligibility. Cronbach's alpha was calculated to assess internal consistency, with a value of 0.82, indicating good reliability.

### 2.3 Data collection

Data was collected through self-administered questionnaires, distributed online via an electronic form (Google Forms); all responses were completed through this online form. Participants were provided with a detailed explanation of the study's purpose and assured of the confidentiality of their responses. Informed consent was obtained from all participants before they completed the questionnaire. The survey data collected was kept anonymous and confidential to ensure participant privacy. To prevent duplication, participants were permitted to complete the questionnaire only once. Additionally, all incomplete questionnaires were excluded from the statistical analysis to maintain data integrity and accuracy.

### 2.4 Statistical analysis

Data was analyzed using SAS 9.4. (SAS Institute Inc., Cary, NC) and a two-sided alpha level <0.05 was considered significant. Correct answers in each section were defined, and internal consistency was assessed using Cronbach alpha. Descriptive statistics were used to summarize demographic characteristics, oral hygiene practices, GR-related factors, and participants' knowledge about GR. Categorical variables were expressed as frequencies and percentages, while continuous variables were expressed as means and standard deviations. Inferential statistics, including chi-square tests and logistic regression analysis, were used to assess associations between variables. A *p*-value <0.05 was considered statistically significant. For each OHIP-14 outcome, the absence of gingival recession served as the reference category, and each item was modelled so that the more favorable OHIP-14 response was the modelled event; consequently, an odds ratio below 1 indicates that gingival recession is associated with lower odds of a favorable response, that is, poorer oral health–related quality of life. Both unadjusted (crude) and adjusted models were fitted. The adjusted models included age, gender, educational level, and socioeconomic status, which were selected *a priori* as recognized sociodemographic determinants of both gingival recession and oral health–related quality of life, so that the association of GR with each outcome could be estimated independently of these factors.

## 3 Results

A total of 619 participants were included in the study. The sample spanned all adult age groups, with the largest single age group comprising 245 participants (39.6%) and a near-equal distribution of males and females; the full age distribution is presented in Table 1. Detailed demographic, socioeconomic, behavioral, and oral-health characteristics of the study population have been comprehensively reported in our previous publication (10) using the same cohort. Our previous paper examined the prevalence of gingival recession, public awareness, and its associated risk factors. The OHIP-14 data analyzed here were collected as a pre-planned component of the same protocol but were neither analyzed nor reported in that publication; the two papers therefore share the cohort and its baseline characteristics while addressing separate, non-overlapping outcomes.

**Table 1:** Chi-square analysis of study variables.

Variable	Category	GR Present *n* (%)	GR Absent *n* (%)	Total *n* (%)	*p*-value
Age	18–29	40 (24.2%)	205 (75.8%)	245 (39.6%)	0.0001*
	30–39	53 (32.3%)	111 (67.7%)	164 (26.5%)	
	40–49	49 (35.8%)	88 (64.2%)	137 (22.1%)	
	50–59	20 (32.3%)	42 (67.7%)	62 (10.0%)	
	≥60	3	8	11 (1.8%)	
Gender	Male	97 (30.4%)	222 (69.6%)	319 (51.5%)	0.0295*
	Female	68 (22.7%)	232 (77.3%)	300 (48.5%)	
Socioeconomic status	Low	72 (20.6%)	277 (79.4%)	349 (56.4%)	0.0006*
	Middle	77 (34.2%)	148 (65.8%)	225 (36.3%)	
	High	16 (35.6%)	29 (64.4%)	45 (7.3%)	
Dental clinic type	Private	65 (26.0%)	185 (74.0%)	250 (40.4%)	0.7613
	Government	100 (27.1%)	269 (72.9%)	369 (59.6%)	
Insurance	Yes	75 (27.6%)	197 (72.4%)	272 (43.9%)	0.6476
	No	90 (25.9%)	257 (74.1%)	347 (56.1%)	
Dental visits	Regular	21 (29.2%)	51 (70.8%)	72 (11.6%)	0.5975
	Irregular	38 (23.8%)	122 (76.3%)	160 (25.8%)	
	Only when in pain	106 (27.4%)	281 (72.6%)	387 (62.5%)	

* *p* < 0.05 showing significant difference.

### 3.1 Bivariate associations between gingival recession and study variables

Several subject characteristics significantly linked to perceived gingival recession (GR) utilizing Chi-square analysis (Table 1). GR prevalence increased significantly and progressively with advancing age (*p*-value = 0.0001), with the highest prevalence observed among individuals aged 40–49 and 50–59 years. Subject's gender also showed a significant link with GR, with males having a greater prevalence of recession compared to females (30.4% vs. 22.7%, *p*-value = 0.0295). Socioeconomic status was similarly influential, participants in the middle- and high-income categories presented with recession more frequently than those in the low-income group (*p*-value = 0.0006). Patterns of dental care utilization (clinic type, medical insurance, and dental visit frequency) showed no significant associations.

Analysis of PROMs revealed that GR was significantly associated with all domains of the OHIP-14 questionnaire. Participants with perceived recession consistently reported more frequent functional limitations, physical discomfort, psychological distress, and social impairment than those without recession. The least affected OHIP-14 item was difficulty with pronunciation, with 72.9% of participants reporting “never” experiencing this problem, while 13.3%, 10.2%, 1.9%, and 1.8% reported experiencing it “hardly ever,” “occasionally,” “fairly often,” and “very often,” respectively. In contrast, painful aching in the mouth was the most frequently reported complaint. Only 15.5% of participants reported “never” experiencing pain, whereas 27.0%, 42.5%, 7.0%, and 8.1% reported pain “hardly ever,” “occasionally,” “fairly often,” and “very often,” respectively. Difficulty eating was also commonly reported (76.4%), indicating that oral conditions may adversely affect dietary habits and overall comfort during meals.

**Figure 1:**
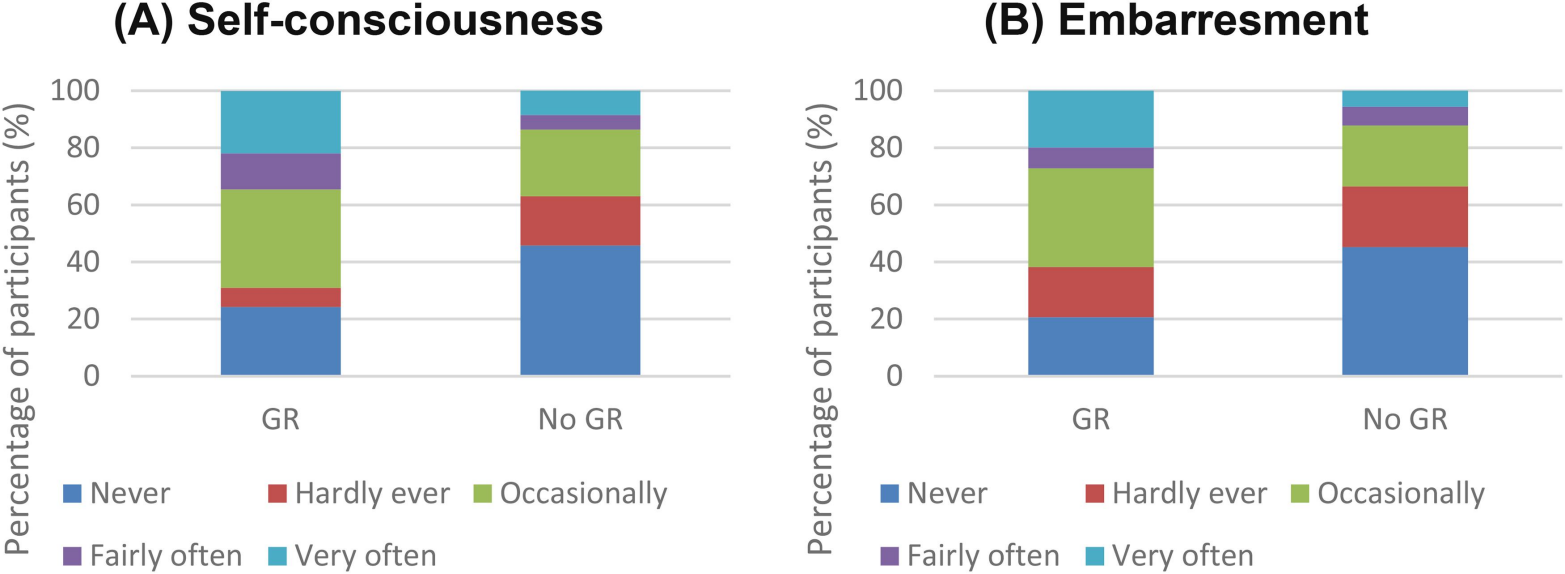
Distribution of psychosocial OHIP-14 response categories by perceived gingival recession status.

The strongest associations were observed within the psychological domains, where self-consciousness and embarrassment were substantially more prevalent among individuals with gingival recession (59.9% and 61.4%, respectively), with Figures 1A,B illustrating a clear shift toward more frequent negative responses specifically higher proportions reporting these impacts “fairly often” or “very often” compared with participants without recession. Although fewer participants reported severe social restrictions, nearly half (48.3%) felt their life satisfaction was reduced, and 33.4% had difficulty performing usual activities. Additionally, participants with GR more frequently experienced diet dissatisfaction, interruptions during meals, irritability, difficulty relaxing, reduced ability to perform daily tasks, diminished life satisfaction, and limitations in overall function (*p*-values <0.0001 and 0.0029, respectively) illustrated in Supplementary Figure S2. Participants were then further categorized into good (<35) and poor (≥35) oral health–related quality of life (OHIP) based on their total OHIP-14 scores, allowing comparison of overall quality-of-life impairment between individuals with or without gingival recession. Within subjects having gingival recession 14.6% were classified as having poor overall (OHIP) score, compared with 2.9% of those without gingival recession. This difference was statistically significant (*χ*^2^ = 29.39, *p* < 0.0001), indicating a substantially greater burden of overall quality-of-life impairment among individuals with gingival recession.

### 3.2 Associations of gingival recession with patient-reported outcomes (PROMs)

The logistic regression analyses were first conducted to examine the unadjusted association between gingival recession (GR) and each of the 14 patient-reported outcome measures (PROMs) derived from the OHIP-14 questionnaire (Table 2). Across all PROMs, the presence of GR was significantly associated with a shift toward more frequent negative oral-health related outcomes. Participants with GR consistently demonstrated lower odds of remaining in a more favorable response categories across domains of functional limitation, physical pain, psychological discomfort, social disability, and unable to properly function. The strongest unadjusted associations were observed for PROMs indicating a psychosocial burden, including self-consciousness **(**OR = 0.309, 95% CI: 0.222–0.430, *p* < 0.0001**)** and embarrassment (OR = 0.320, 95% CI: 0.231–0.445, *p* < 0.0001**).** PROMs related to pain, difficulty with eating, and interruption of daily activities also demonstrated significant negative associations. An adjusted logistic regression models were subsequently performed for each PROM outcome, accounting for potential confounders after adjustment for age, gender, educational level, and socioeconomic status (Table 3).

**Table 2:** Unadjusted associations between gingival recession and OHIP-14 outcomes.

PROM outcome	Unadjusted OR	95% CI	*P*-Value
Pronunciation	0.394	0.272–0.571	<0.0001*
Taste	0.509	0.353–0.733	0.0003*
Pain	0.404	0.288–0.565	<0.0001*
Eat	0.399	0.288–0.554	<0.0001*
Consciousness	0.309	0.222–0.430	<0.0001*
Tension	0.337	0.243–0.467	<0.0001*
Diet	0.347	0.249–0.483	<0.0001*
Interruption	0.477	0.345–0.660	<0.0001*
Relax	0.430	0.310–0.596	<0.0001*
Embarrassed	0.320	0.231–0.445	<0.0001*
Irritable	0.374	0.267–0.525	<0.0001*
Job	0.489	0.351–0.681	<0.0001*
Life Unsatisfying	0.372	0.267–0.518	<0.0001*
Unable to Function	0.516	0.362–0.736	0.0003*

OR, odds ratio; CI, confidence interval. Gingival recession was coded as 1 = present and 2 = absent (reference category). OHIP-14 response categories were coded from 1 = never to 5 = very often. Odds ratios below 1 indicate a greater likelihood of reporting more frequent negative oral health impacts among participants with gingival recession compared with those without gingival recession.

* *p* < 0.05 indicates statistical significance.

**Table 3:** Adjusted associations between gingival recession and OHIP-14 outcomes.

PROM outcome	Adjusted OR	95% CI	*P*-value
Pronunciation	0.383	0.254–0.576	<0.0001*
Taste	0.518	0.348–0.771	0.0012*
Pain	0.452	0.317–0.644	<0.0001*
Eat	0.423	0.299–0.598	<0.0001*
Consciousness	0.328	0.231–0.466	<0.0001*
Tension	0.351	0.248–0.497	<0.0001*
Diet	0.362	0.254–0.516	<0.0001*
Interruption	0.521	0.368–0.738	0.0002*
Relax	0.454	0.321–0.642	<0.0001*
Embarrassed	0.327	0.230–0.465	<0.0001*
Irritable	0.409	0.248–0.497	<0.0001*
Job	0.560	0.393–0.799	<0.0001*
Life Unsatisfying	0.399	0.280–0.568	<0.0001*
Unable to Function	0.528	0.358–0.778	0.0012*

Adjusted for age, gender, educational level, and socioeconomic status. OR, odds ratio; CI, confidence interval. Gingival recession was coded as 1 = present and 2 = absent (reference category). OHIP-14 response categories were coded from 1 = never to 5 = very often. Odds ratios below 1 indicate a greater likelihood of reporting more frequent negative oral health impacts among participants with gingival recession compared with those without gingival recession.

* *p* < 0.05 indicates statistical significance.

**Figure 2:**
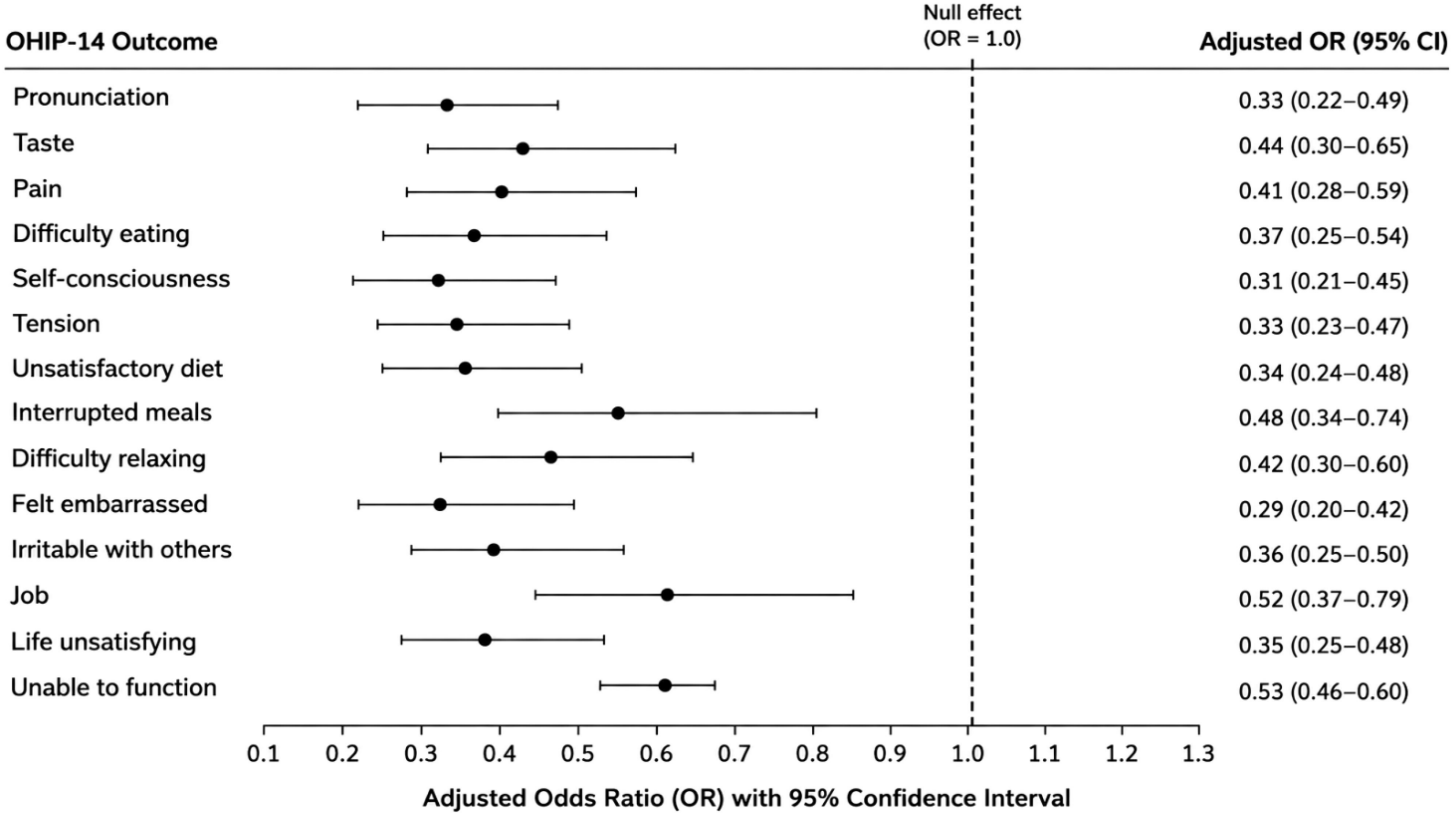
Adjusted associations between gingival recession and OHIP-14 patient-reported outcome measures. Forest plot showing adjusted odds ratios (ORs) and 95% confidence intervals (CIs) for the association between perceived gingival recession and individual OHIP-14 patient-reported outcome measures. Estimates were obtained from multivariable ordinal logistic regression models adjusted for age, gender, educational level, and socioeconomic status. Dots represent adjusted ORs and horizontal lines represent the corresponding 95% CIs. The vertical dashed line indicates the null value (OR = 1.0), representing no association. Odds ratios below 1 indicate a greater likelihood of reporting more frequent negative oral health-related quality-of-life impacts among participants with perceived gingival recession compared with those without perceived gingival recession.

GR was consistently significantly associated with worse outcomes across all 14 PROMs, with adjusted odds ratios below 1.0 for all items (Table 3). The magnitude and direction of the adjusted associations between gingival recession and all OHIP-14 patient-reported outcomes are visually summarized in Figure 2. This forest plot complements Table 3 by displaying the adjusted odds ratios and their 95% confidence intervals for all 14 items on a single axis, allowing the size, direction, and precision of each association to be compared easily.

## 4 Discussion

This cross-sectional study assessed the association between gingival recession (GR) and oral health–related quality of life (OHRQoL) among adults in Saudi Arabia using a modified version of the OHIP-14 instrument. Findings from our study demonstrated that gingival recession was significantly associated with poorer patient-reported outcomes across multiple OHIP-14 domains. These findings are consistent with those reported by Wagner et al., who observed worse oral health-related quality of life among individuals with gingival recession (15). Domains such as physical pain, psychological discomfort, difficulty eating, and reduced life satisfaction were the most affected, supporting the assumption that oral disease extends far beyond physical symptoms to influence emotional well-being and social function. These findings align with the World Health Organization's definition of oral health as not merely the absence of disease but a state of overall physical and social well-being (16).

A considerable proportion of participants reported functional limitations associated with GR, including difficulty eating, speaking, and maintaining normal oral function. More than 70% of participants reported pain or sensitivity during eating, and nearly one-quarter indicated difficulty pronouncing words. Similar trends were reported in different Arab countries, where oral discomfort interfered with daily mastication and communication, particularly among individuals with untreated periodontal issues (12, 17). The high functional impact reported in our study suggests that patients often endure discomfort rather than actively seeking dental care. This may reflect a lack of awareness regarding treatment options, a pattern also observed in other Middle Eastern populations where dental visits remain largely symptom-driven rather than preventive (18).

Physical pain emerged as the most frequently reported OHIP-14 domains, with painful aching and sensitivity affecting more than 80% of participants at least occasionally. A consensus-based report established that gingival recession with exposed root surfaces is primary etiological factor for dentine hypersensitivity (19). This biological relationship may help explain the observed association between GR and the high prevalence of pain and sensitivity observed among participants with gingival recession in the present study.

Psychological discomfort was also prominent in this study, with more than half of the participants reporting embarrassment, self-consciousness, or difficulty relaxing as a result of oral problems reflecting their psychosocial burden. Similar findings have been reported previously, where gingival recession and root exposure were associated with aesthetic concerns that negatively affected self-esteem and oral health–related quality of life (12, 20, 21). The psychosocial impact of gingival recession may be particularly pronounced when recession affects the anterior esthetic region, where visible changes in gingival appearance can influence self-perception and social confidence (14, 22–24). Studies have shown that individuals with visible gingival recession may avoid smiling or social interactions because of embarrassment and dissatisfaction with their appearance (25). Taken together, these observations indicate that the association with gingival recession extends beyond functional impairment and includes important psychological and esthetic consequences.

In the present study, males had the highest reported GR prevalence, which may reflect differences in oral hygiene behaviors, and utilization of preventive dental services. This observation is consistent with findings from other studies, including a study from Saudi Arabia, which identified male sex as a significant risk indicator for GR (26, 27), although those studies employed clinical examination rather than self-reported perception.

The findings revealed that many participants had insufficient preventive oral hygiene habits, such as irregular brushing or inappropriate brushing techniques. One-third of subjects reported utilizing a random/mixed brushing technique, and around 20% reported brushing with a horizontal technique. Aggressive horizontal brushing and the use of hard-bristled toothbrushes are known risk factors for the progression of GR (28). Most subjects (62%) reported only going to the dentist when in pain, which is in line with results reported by Nermo et al. (29); irregular dental visits were associated with more frequent reports of pain and functional limitation, supporting the known association between periodontal health and health-seeking behavior. These behavioral findings reinforce the need for structured patient education and early intervention strategies (30–32).

The level of impact on quality of life observed in this study may also reflect cultural and social influences. In many Middle Eastern societies, dental esthetics and personal presentation are increasingly valued, and oral health issues may have a stronger psychosocial effect than in Western populations (18, 33). However, dental health awareness remains low, and misconceptions about GR being a normal sign of aging persist (34). Public health strategies must therefore integrate culturally sensitive oral health awareness campaigns. Dental service utilization variables, including frequency of dental visits, type of dental clinic, and insurance coverage, were not significantly associated with gingival recession in the present study. This contrasts with findings reported by Beigi et al., who demonstrated that individuals with basic insurance had significantly fewer dental visits, highlighting the impact of insurance coverage on patients' willingness to seek dental care (35). These findings may reflect a predominantly problem-oriented pattern of dental attendance, in which patients seek care primarily for symptomatic issues rather than preventive periodontal evaluation. As a result, gingival recession may remain undiagnosed or unmanaged until it becomes clinically or esthetically concerning.

Socioeconomic factors were also associated with gingival recession in the present study, with a higher prevalence observed among individuals from middle- and higher-income groups. This finding may reflect greater esthetic awareness and reporting accuracy, as well as potentially more aggressive oral hygiene practices. Similar observations have been reported previously, where gingival recession was more prevalent among individuals with higher education and income levels (24, 36). One proposed explanation is that individuals seeking to maintain optimal oral health may inadvertently adopt traumatic oral hygiene practices that contribute to gingival recession (36).

Adjusted logistic regression models confirmed that gingival recession remained a significant independent predictor of worse outcomes across all 14 OHIP-14 items after controlling for age, gender, education, and socioeconomic status. The adjusted odds ratios were consistently below 1.0 and comparable in magnitude to the unadjusted estimates, with no meaningful attenuation. This stability indicates that the negative impact of gingival recession on patient-reported oral health cannot be explained by demographic or socioeconomic differences alone, reflecting a true independent association across functional, physical, and psychosocial domains.

The findings highlight that GR should not be underestimated clinically. This condition has traditionally been regarded as a minor periodontal problem; however, the present findings demonstrate significant interference with daily comfort and emotional well-being. Dentists should proactively identify early signs of GR, screen for quality-of-life impact, and adopt minimally invasive treatment approaches. Multidimensional patient care addressing both symptoms and quality-of-life domains is recommended. Preventive strategies, patient education, and incorporation of patient-reported outcomes into routine periodontal assessment may enhance both clinical care and patient-centered treatment planning.

Several limitations should be acknowledged. The cross-sectional design precludes causal inference. Also, the reliance on self-reported gingival recession without clinical verification by a dental professional. Although participants were provided with visual aid and written description of the condition prior to completing the survey, self-assessment may still lead to misclassification due to overestimation or underestimation of recession. Self-report also could not capture the severity or extent of recession or distinguish localized from generalized forms. In addition, the OHIP-14 is a generic measure of oral health-related quality of life rather than a recession-specific instrument: it reflects the overall impact of oral conditions on daily functioning, so the observed associations may partly reflect conditions that commonly accompany gingival recession, such as periodontitis, dentine hypersensitivity, and cervical tooth wear, rather than recession alone. Finally, the good/poor OHIP-14 threshold is a pragmatic cut-point rather than a universally validated one, and recruitment relied on a non-probabilistic, online convenience sample, which may introduce selection bias and limits how far the findings can be generalized to the wider Saudi adult population. Nevertheless, the large sample size, comprehensive sociodemographic analysis, and robust evaluation of quality-of-life outcomes strengthen the validity and relevance of the findings. Longitudinal studies are needed to evaluate causality and changes in quality of life after treating gingival recession. Additionally, randomized controlled trials could examine whether educational or therapeutic interventions improve OHRQoL in patients with GR.

## Conclusion

This study found that self-reported gingival recession is significantly associated with poorer oral health–related quality of life among adults in Saudi Arabia, particularly in terms of physical pain, daily functional limitations, and psychological discomfort. These findings highlight the importance of early diagnosis, preventive care, and comprehensive management strategies. Public awareness programs and integration of patient-reported outcomes in routine dental assessments are recommended to improve quality of life outcomes. Because recession was based on participant self-report rather than clinical examination, these conclusions describe self-perceived recession and should be confirmed in studies that include calibrated clinical assessment.

## Data Availability

The original contributions presented in the study are included in the article/Supplementary Material, further inquiries can be directed to the corresponding author.
