# Supplementary material for: The impact of perceived gingival recession on oral health-related quality of life: a cross-sectional study of Saudi adults
Source: Front Oral Health. 2026 Jul 15;7:1878291. doi: 10.3389/froh.2026.1878291 (PMC13415773; doi:10.3389/froh.2026.1878291)
Supplement: Supplementary file 1 [file Datasheet1.docx]

**OHIP-14**

Dear Participant,

Thank you for agreeing to take part in this research study. The aim of this study is to assess the awareness and perception of gingival recession (gum shrinkage) and its impact on daily life and oral health among adults in Saudi Arabia.

**What is gingival recession?** Gingival recession occurs when the gum tissue surrounding the teeth pulls back or wears away, exposing more of the tooth or the tooth's root. This may cause the teeth to appear longer than normal, and you may notice a gap or change in color or a "step" between the gum line and where it used to be. In some cases, the exposed root surface may feel sensitive to hot, cold, or sweet foods and drinks. Please refer to the photograph below for an example of how gingival recession may appear.


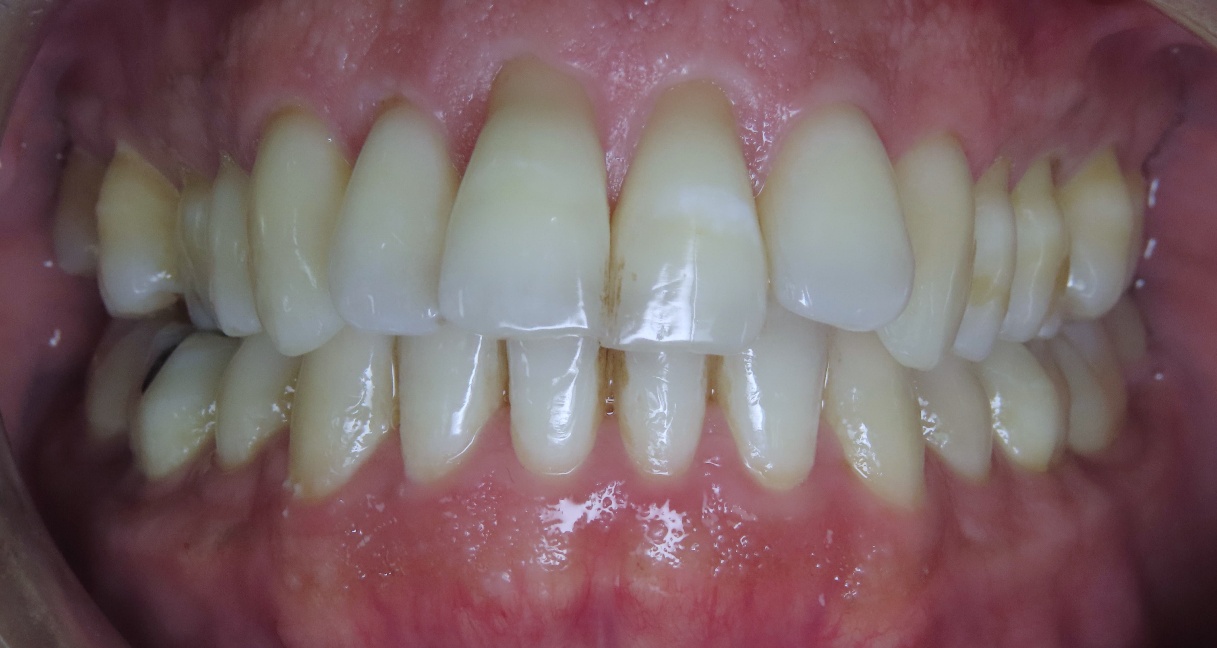


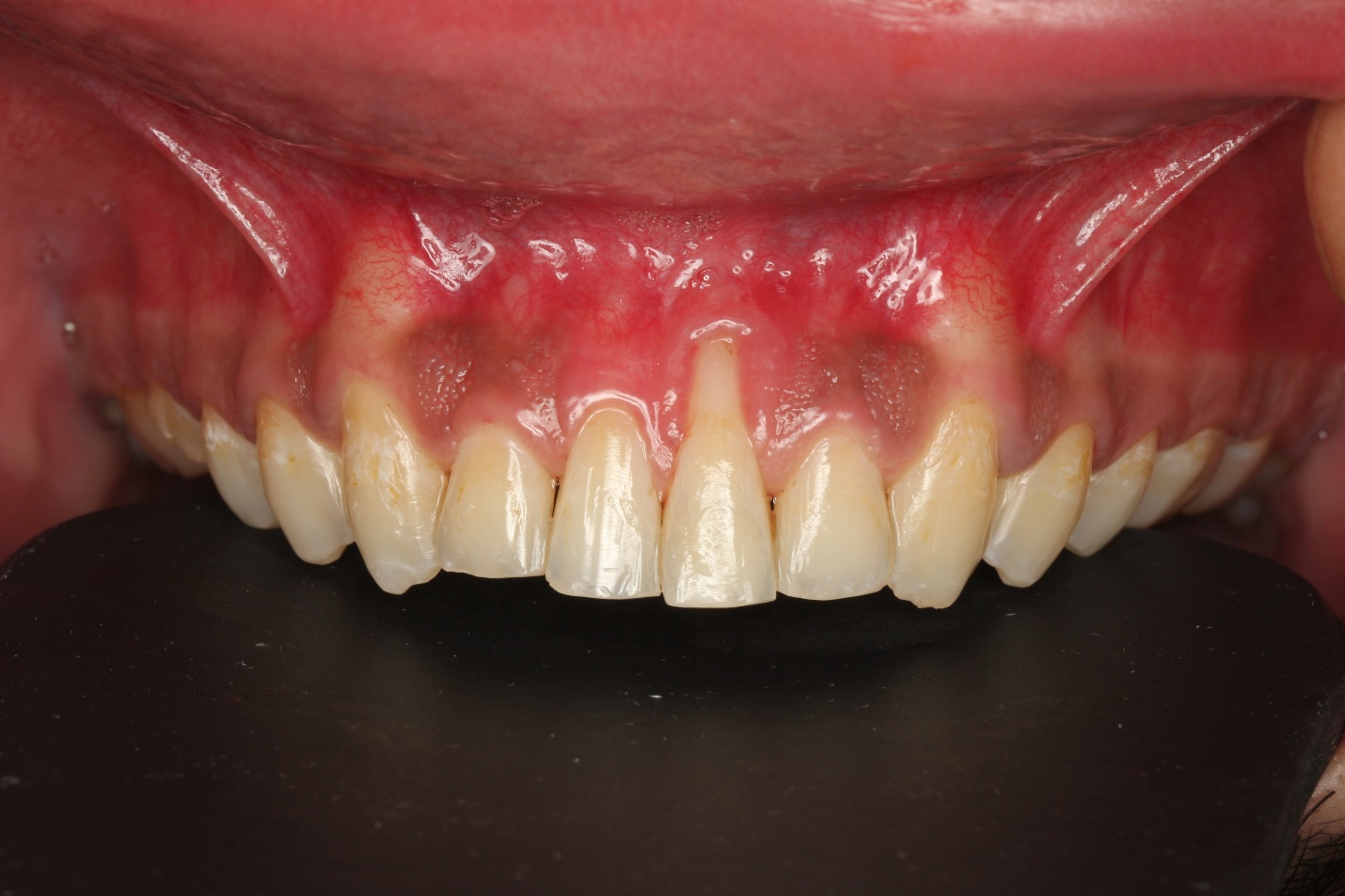


Your participation is voluntary and all responses will be kept strictly confidential and anonymous. The questionnaire will take approximately 5–10 minutes to complete. By proceeding, you consent to participate in this study.

Questions for the OHIP-14

| Question no. |  | 0 = never | 1=hardly ever | 2= occasionally | 3= fairly often | 4=very often |
| --- | --- | --- | --- | --- | --- | --- |
| 1 | Have you had trouble pronouncing any words because of problems with limitation your teeth, mouth or dentures? |  |  |  |  |  |
| 2 | Have you felt that your sense of taste has worsened because of problems with your teeth, mouth or dentures? |  |  |  |  |  |
| 3 | Have you had painful aching Physical in your mouth? |  |  |  |  |  |
| 4 | Have you found it uncomfortable to eat any foods because of problems with your teeth, mouth or dentures? |  |  |  |  |  |
| 5 | Have you been self-conscious because of your teeth, mouth or dentures? |  |  |  |  |  |
| 6 | Have you felt tense because of problems with your teeth, mouth or dentures? |  |  |  |  |  |
| 7 | Has your diet been unsatisfactory because of problems with your teeth, mouth or dentures? |  |  |  |  |  |
| 8 | Have you had to interrupt meals because of problems with your teeth, mouth or dentures? |  |  |  |  |  |
| 9 | Have you found it difficult to relax because of problems with your teeth, mouth or dentures? |  |  |  |  |  |
| 10 | Have you been a bit embarrassed because of problems with your teeth, mouth or dentures? |  |  |  |  |  |
| 11 | Have you been a bit irritable with other people because of problems with your teeth, mouth or dentures? |  |  |  |  |  |
| 12 | Have you had difficulty doing your usual jobs because of problems with your teeth, mouth or dentures? |  |  |  |  |  |
| 13 | Have you felt that life in general was less satisfying because of problems with your teeth, mouth or dentures? |  |  |  |  |  |
| 14 | Have you been totally unable to function because of problems with your teeth, mouth or dentures? |  |  |  |  |  |

End of questions

Thank you

عزيزي المشارك / عزيزتي المشاركة

نشكرك على موافقتك على المشاركة في هذه الدراسة البحثية. تهدف هذه الدراسة إلى تقييم مدى الوعي والإدراك بانحسار اللثة (تراجع اللثة) وتأثيره على الحياة اليومية وصحة الفم لدى البالغين في المملكة العربية السعودية

**ما هو انحسار اللثة؟** يحدث انحسار اللثة عندما تتراجع أنسجة اللثة المحيطة بالأسنان أو تتآكل، مما يؤدي إلى كشف جزء أكبر من السن أو جذر السن. قد يُلاحَظ أن الأسنان تبدو أطول من المعتاد، وقد تظهر فجوة اوتغيىر في اللون أو "درجة" بين خط اللثة الحالي وموقعه السابق. في بعض الحالات، قد يشعر الشخص بحساسية في المنطقة المكشوفة عند تناول الأطعمة أو المشروبات الساخنة أو الباردة أو الحلوة. يرجى الاطلاع على الصورة أدناه لمعرفة كيف يبدو انحسار اللثة


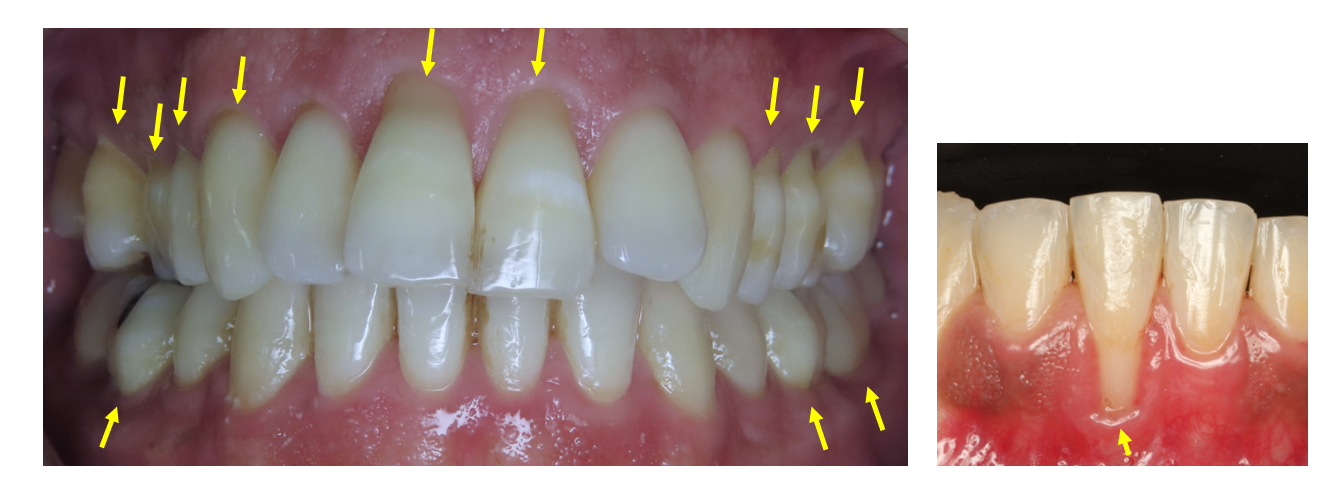


مشاركتك في هذه الدراسة طوعية، وستبقى جميع إجاباتك سرية تمامًا ومجهولة الهوية. يستغرق ملء الاستبيان حوالي ٥–١٠ دقائق. بمتابعتك للإجابة، فإنك توافق على المشاركة في هذه الدراسة.

أسئلة الملف التعريفي لتأثير صحة الفم(OHIP-14)

| م. |  | 0 = أبدا | 1 = نادرا | 2 = أحيانًا | 3 = في كثير من الأحيان إلى حد ما | 4= في كثير من الأحيان |
| --- | --- | --- | --- | --- | --- | --- |
| ١ | هل واجهت صعوبة في نطق أي كلمات بسبب مشاكل في تقييد أسنانك أو فمك أو أطقم الأسنان؟ |  |  |  |  |  |
| ٢ | هل شعرت أن حاسة التذوق لديك قد ساءت بسبب مشاكل في أسنانك أو فمك أو أطقم الأسنان؟ |  |  |  |  |  |
| ٣ | هل عانيت من آلام جسدية مؤلمة في فمك؟ |  |  |  |  |  |
| ٤ | هل وجدت أنه من غير المريح تناول أي أطعمة بسبب مشاكل أسنانك أو فمك أو أطقم الأسنان؟ |  |  |  |  |  |
| ٥ | هل كنت خجولًا بسبب أسنانك أو فمك أو أطقم الأسنان؟ |  |  |  |  |  |
| ٦ | هل شعرت بـتوتربسبب مشاكل في أسنانك أو فمك أو أطقم الأسنان؟ |  |  |  |  |  |
| ٧ | هل كان نظامك الغذائي غير مرضٍ بسبب مشاكل في أسنانك أو فمك أو أطقم الأسنان؟ |  |  |  |  |  |
| ٨ | هل اضطررت إلى مقاطعة وجبات الطعام بسبب مشاكل في أسنانك أو فمك أو أطقم الأسنان؟ |  |  |  |  |  |
| ٩ | هل وجدت صعوبة في الاسترخاء بسبب مشاكل في أسنانك أو فمك أو أطقم الأسنان؟ |  |  |  |  |  |
| ١٠ | هل شعرت بالحرج قليلاً بسبب مشاكل في أسنانك أو فمك أو أطقم الأسنان؟ |  |  |  |  |  |
| ١١ | هل كنت سريع الانفعال قليلاً مع أشخاص آخرين بسبب مشاكل في أسنانك أو فمك أو أطقم الأسنان؟ |  |  |  |  |  |
| ١٢ | هل واجهت صعوبة في أداء وظائفك المعتادة بسبب مشاكل في أسنانك أو فمك أو أطقم الأسنان؟ |  |  |  |  |  |
| ١٣ | هل شعرت أن الحياة بشكل عام كانت أقل إرضاءً بسبب مشاكل أسنانك أو فمك أو أطقم الأسنان؟ |  |  |  |  |  |
| ١٤ | هل عجزت تمامًا عن العمل بسبب مشاكل في أسنانك أو فمك أو أطقم الأسنان؟ |  |  |  |  |  |

نهاية الأسئلة

شكرا لك
