# Supplementary material for: The impact of perceived gingival recession on oral health-related quality of life: a cross-sectional study of Saudi adults
Source: Front Oral Health. 2026 Jul 15;7:1878291. doi: 10.3389/froh.2026.1878291 (PMC13415773; doi:10.3389/froh.2026.1878291)
Supplement: Supplementary file 2 [file Datasheet2.docx]

**Supplemental Figure S2: Distribution of OHIP-14 response categories by perceived gingival recession status.**
